# High-permeability region size on perfusion CT predicts hemorrhagic transformation after intravenous thrombolysis in stroke

**DOI:** 10.1371/journal.pone.0188238

**Published:** 2017-11-28

**Authors:** Josep Puig, Gerard Blasco, Pepus Daunis-i-Estadella, Cecile van Eendendburg, María Carrillo-García, Carlos Aboud, María Hernández-Pérez, Joaquín Serena, Carles Biarnés, Kambiz Nael, David S. Liebeskind, Götz Thomalla, Bijoy K. Menon, Andrew Demchuk, Max Wintermark, Salvador Pedraza, Mar Castellanos

**Affiliations:** 1 Research Unit of Diagnostic Imaging Institute (IDI), Department of Radiology [Girona Biomedical Research Institute] IDIBGI, Hospital Universitari Dr Josep Trueta, Girona, Spain; 2 Department of Computer Science, Applied Mathematics and Statistics, University of Girona, Girona, Spain; 3 Department of Neurology-IDIBGI, Dr Josep Trueta University Hospital, Girona, Spain; 4 Department of Radiology, Morales Meseguer University Hospital, Murcia, Spain; 5 Department of Radiology, La Fe University Hospital, Valencia, Spain; 6 Stroke Unit, Germans Trias i Pujol University Hospital, Badalona, Spain; 7 Department of Radiology, Icahn School of Medicine at Mount Sinai, New York, New York, United States of America; 8 Neurovascular Imaging Research Core and UCLA Stroke Center, Los Angeles, United States of America; 9 Department of Neurology, University Medical Centre Hamburg-Eppendorf, Hamburg, Germany; 10 Calgary Stroke Program, Hotchkiss Brain Institute, University of Calgary, Canada, Alberta, Canada; 11 Department of Radiology, Neuroradiology Division, Stanford University, Palo Alto, California, United States of America; 12 Department of Neurology, A Coruña University Hospital, Biomedical Research Institute, A Coruña, Spain; Henry Ford Health System, UNITED STATES

## Abstract

**Objective:**

Blood-brain barrier (BBB) permeability has been proposed as a predictor of hemorrhagic transformation (HT) after tissue plasminogen activator (tPA) administration; however, the reliability of perfusion computed tomography (PCT) permeability imaging for predicting HT is uncertain.

We aimed to determine the performance of high-permeability region size on PCT (HP_rs_-PCT) in predicting HT after intravenous tPA administration in patients with acute stroke.

**Methods:**

We performed a multimodal CT protocol (non-contrast CT, PCT, CT angiography) to prospectively study patients with middle cerebral artery occlusion treated with tPA within 4.5 hours of symptom onset. HT was graded at 24 hours using the European-Australasian Acute Stroke Study II criteria. ROC curves selected optimal volume threshold, and multivariate logistic regression analysis identified predictors of HT.

**Results:**

The study included 156 patients (50% male, median age 75.5 years). Thirty-seven (23,7%) developed HT [12 (7,7%), parenchymal hematoma type 2 (PH-2)]. At admission, patients with HT had lower platelet values, higher NIHSS scores, increased ischemic lesion volumes, larger HP_rs_-PCT, and poorer collateral status. The negative predictive value of HP_rs_-PCT at a threshold of 7mL/100g/min was 0.84 for HT and 0.93 for PH-2. The multiple regression analysis selected HP_rs_-PCT at 7mL/100g/min combined with platelets and baseline NIHSS score as the best model for predicting HT (AUC 0.77). HP_rs_-PCT at 7mL/100g/min was the only independent predictor of PH-2 (OR 1, AUC 0.68, p = 0.045).

**Conclusions:**

HP_rs_-PCT can help predict HT after tPA, and is particularly useful in identifying patients at low risk of developing HT.

## Introduction

Hemorrhagic transformation (HT) can be one of the most devastating complications of acute ischemic stroke. HT arises when ischemia-induced vascular damage that disrupts the blood-brain barrier (BBB) is followed by reperfusion [1]. Revascularization with tissue plasminogen activator (tPA) increases the risk of HT 10-fold [2]. Clinical guidelines restrict tPA administration to the first 4.5 h after onset [3], and potential medicolegal concerns about HT lead to underuse of tPA. Factors associated with increased risk of HT include age, baseline National Institute of Health Stroke Scale (NIHSS) score, previous antithrombotic treatment, and thrombolytic treatment [4]. Imaging parameters associated with increased risk of HT include early ischemic changes on non-contrast computed tomography (CT) quantified using the Alberta Stroke Program Early CT Score (ASPECTS), leukoaraiosis, cerebral blood volume (CBV) at admission, ischemic lesion volume derived from mean transit time (MTT) maps, large-vessel occlusions, and poor collaterals on CT angiography (CTA) [5–9], but no method to predict the development of HT after intravenous tPA has been clinically approved.

Accurate methods for classifying patients’ risk of HT would make tPA administration within the current 4.5-hour time-window safer and might enable more patients to benefit from tPA beyond this window. One approach to identifying patients at risk of HT is to seek evidence of BBB damage before thrombolysis, In this respect, a promising method to determine BBB permeability consists of modeling the transfer constant (*K*^trans^), a metric of BBB integrity, based on the measurement of the intravascular tracer leakage into the extravascular space [10,11]. In the normal brain, the BBB is impermeable to the relatively large hydrophilic molecules in iodinated contrast agents, so *K*^trans^ = 0. A damaged BBB results in *K*^trans^>0, and nonzero values represent diffusion across a damaged membrane quantitatively. Commercially available software can semi-automatically generate a functional color map of the brain permeability.

Growing evidence suggests that BBB permeability can increase in the first few hours after stroke and that abnormally elevated parameters on perfusion CT (PCT)-derived permeability maps might indicate ischemia-induced vascular damage [12] and serve as markers to predict HT [13–19]. However, this approach has not been incorporated into standard of care, in part due to concerns regarding its sensitivity, specificity, and predictive values. Since the prediction of HT, and especially PH2, is essential to improve the ratio risk/benefit in patients receiving tPA, we aimed to determine the performance of high-permeability region size on PCT (HP_rs_-PCT) at admission in predicting HT after tPA administration.

## Methods

The Ethics Committee of Hospital Universitari Dr. Josep Trueta of Girona approved this study.

### Patients

This is a prospective study of patients with acute stroke treated with tPA at a university hospital between October 2012 and June 2016. For the purpose of this study, inclusion criteria were as follows: (1) first-ever middle cerebral artery (MCA) territory infarction treated with tPA within 4.5 hours of symptom onset, (2) multimodal PCT performed at admission and (3) non-contrast CT follow-up at 24 hours. Patients presenting motion artifacts were excluded. Patients without clinical improvement after tPA who underwent mechanical thrombectomy were also excluded. Stroke severity was assessed by using the NIHSS score before tPA administration and immediately after the end of the infusion, and at 24, 48 and 72 hours of evolution. Functional outcome was evaluated by using the modified Rankin Scale (mRS) at 3 months; patients were considered to have achieved a good functional outcome if mRS was ≤2. Stroke mechanism was classified as large artery atherosclerosis, cardioembolic, and of undetermined cause following the modified TOAST criteria [20].

### Imaging protocol

All examinations were performed on a 128-slice CT scanner (Ingenuity; Philips Healthcare, Best, the Netherlands). We used the following imaging parameters: a) for noncontrast CT: helical scanning mode, 120kVp, 370mAs, 3-mm slice thickness, 1.5-mm gap, and 512x512 matrix; b) for PCT: cine scanning mode, 100kVp, 100mAs, and four 10-mm slices centered on the basal ganglia. Image acquisition began 7 seconds after starting injection of a 50-mL bolus of iodinated contrast agent (Ultravist 300mgI/mL; Bayer HealthCare Pharmaceutical Inc., Leverkusen, Germany) into an antecubital vein at 5 mL/second with a power injector. We acquired first-pass (25 scans, cycle time 2 seconds) and delayed-phase (six contiguous scans, cycle time 30 seconds) images. There were 3 time frames with an interval of 2 seconds before bolus arrival. c) For CTA (from aortic arch to cranial vertex): helical scanning mode, 120kVp, 250mAs, 0.9-mm slice thickness, 0.45-mm gap, and 512x512 matrix. We acquired CTA images after an 80-mL bolus of Ultravist (300mgI/mL) injected at 5 mL/second followed by 20 mL of saline solution.

#### Image postprocessing

**Fig 1:**
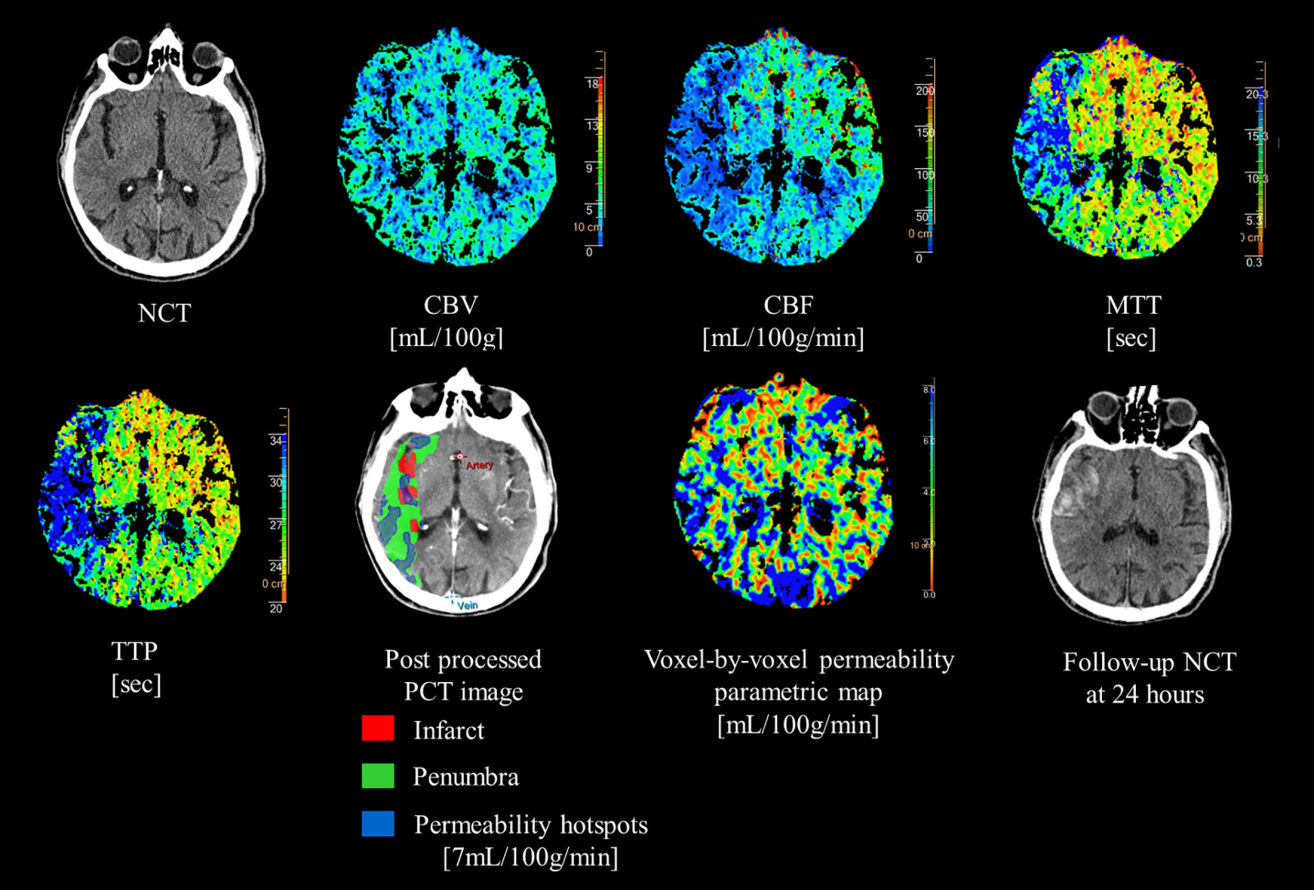
Perfusion CT protocol in a patient with PH-2 in the ischemic right MCA territory following intravenous thrombolysis. Post-processed map shows a large penumbra in right MCA territory. Note the clearly increased baseline permeability at thresholds of 7 ml/100g/min in the area of the parenchymal hematoma.

CBV, cerebral blood flow (CBF), MTT, and time to peak (TTP) were automatically calculated from PCT data using commercial software (Extended Brilliance workstation 4.5, Philips Healthcare). Three manually placed regions of interest were traced in the anterior cerebral artery. The region with the highest enhancement was used as the reference arterial input function and the superior sagittal sinus as the venous output function. The infarct core is by default defined as CBV < 2 ml/100g, and the penumbra as MTT > 145% and CBV ≥ 2 ml/100g. [9] BBB permeability was calculated with commercial software based on the Patlak model (Intellispace Portal system, Philips Healthcare). The Patlak model uses linearized regression for estimating the *K*^trans^ and the blood volume, and it involves fitting regression lines to observations of time-density curves for each pixel and for intravascular reference function. The slopes of these lines represent local blood-to-brain transfer constants that indicate BBB permeability (*K*^trans^ in mL/100g/min). It is important to mention that the leakage is irreversible, and the attenuation curves are in a near steady-state. We applied different thresholds on *K*^trans^ in order to calculate the HP_rs_-PCT. These automatic calculations set the threshold for generating five user-independent permeability maps (at 3, 4, 5, 6 and 7 mL/100g/min) (Fig 1). Anatomic locations normally lacking a BBB (e.g., the ventricular choroid plexuses) often have a high permeability, so they are excluded.

CTA source images were reconstructed to 20-mm-thick axial and coronal maximum intensity projections using Starviewer software (http://gilab.udg.edu/) [21]. To assess vascular status, we used a modified TIMI grading system, as follows: complete occlusion with no perfusion past the thrombus (TIMI 0); perfusion past the initial occlusion without opacification of distal vessels (TIMI 1); partial occlusion (TIMI 2), defined as an obstruction with opacification of the distal vessels; and complete recanalization (TIMI 3). Recanalization was defined as TIMI 2–3 flow in vessels from 1 hour after thrombolysis by using CTA. Collaterals were assessed with the American Society of Interventional and Therapeutic Neuroradiology/Society of Interventional Radiology (ASITN/SIR) Collateral Flow Grading System on CTA [22]. The presence of HT was determined by follow-up CT 24 hours after initial imaging and graded using the European-Australasian Acute Stroke Study II system into 4 subtypes: hemorrhagic infarct type 1 (HI-1), hemorrhagic infarct type 2 (HI-2), parenchymal hematoma type 1 (PH-1), or parenchymal hematoma type 2 (PH-2) [23]. Two neuroradiologists (with over 15 years of experience) blinded to the clinical data jointly evaluated the vascular status and HT. HT was considered as symptomatic (sICH) if the patient had clinical deterioration causing an increase of ≥4 points on the NIHSS [24].

### Statistical analysis

Patients were classified into 2x2 groups: ‘without HT’ vs ‘with HT’ and ‘without PH-2’ vs ‘with PH-2’, further classified as symptomatic (sICH) and non-symptomatic (non-sICH). Results are shown as medians (interquartile ranges) for continuous variables and percentages for categorical variables. To compare clinical and imaging data between groups, we used univariate analyses: Fisher’s exact test for categorical data and the Mann-Whitney test for continuous data. For each outcome, we performed a univariate logistic regression analysis including all variables collected; we then entered variables significant at P<0.05 into a multivariate logistic regression model constructed using the backward elimination method. Variables with P<0.05 in the multivariate analyses were considered independent predictors. Odds ratios (OR) with 95% confidence intervals were calculated. Receiver-operating characteristics (ROC) curves were used to determine the best HP_rs_-PCT cutoffs for each threshold and associated area under curve (AUC) and their sensitivity, specificity, positive predictive value (PPV), and negative predictive value (NPV). We used Minitab version 16.1.1 (Minitab Inc, State College, PA, USA) for all analyses.

## Results

### Patient characteristics

Of 357 consecutive tPA-treated patients, 67 were treated beyond 4.5 hours after stroke onset, 23 did not have a non-contrast CT follow-up at 24 hours, 37 did not have PCT at admission, 14 had a posterior cerebral artery infarction, 6 had a anterior cerebral artery infarction, 23 had a vertebrobasilar territory infarction, and 31 patients underwent mechanical thrombectomy after tPA treatment. Therefore, 156 patients finally met the inclusion criteria. Table 1 summarizes patient characteristics, imaging data, and HP_rs_-PCT measurements at different thresholds. Among the patients included in the study, HT occurred in 37 (23.7%): 10 had HI-1, 7 HI-2, 8 PH-1, and 12 PH-2 (3 with sICH). CBV values did not differ between patients with HT and those without. Patients with HT had lower platelet count, lower fibrinogen values and higher NIHSS scores at admission, at 24 hours, and at 72 hours. They also had higher abnormal MTT volumes, larger HP_rs_-PCT, poorer collateral status, and worse functional outcome at 90 days.

**Table 1:** Patient characteristics and univariate statistics between patients with and without PH-2.

	Without PH-2 (n = 144)	With PH-2 (n = 12)	p-value
**Demographics**			
Age(years)	75(66–81.25)	78.5(69.75–81.25)	0.367
Female,%	47.9	75	0.071
**Risk factors**			
Systolic blood pressure(mm Hg)	145(135–154.5)	142.5(129–168.5)	0.695
Diastolic blood pressure(mmHg)	74(66–83)	78.5(64.25–85.5)	0.429
Hypertension,%	70.1	75.0	0.723
Diabetes,%	20.8	16.7	0.731
Smoking,%	18.8	0.0	0.158
Hyperlipidemia,%	38.9	33.3	0.704
Atrial fibrillation,%	31.2	41.7	0.458
Glucose(mmol/L)	128(108–147)	136(106.5–169)	0.814
Fibrinogen(mg/dL)	416(358–481)	341(310–419.5)	0.063
Platelets(x103)	210.5(172–255.5)	178(170–202)	0.086
**Stroke etiology**			
Large artery,%	15.1	9.1	0.835
Cardioembolic,%	47.5	54.5	
Undetermined/other,%	37.4	36.4	
**Clinical Data**			
NIHSS at admission	13(7–19)	14.5(9–19.25)	0.409
NIHSS at 24 hours	6(2–17.25)	11(6.5–19.5)	0.113
NIHSS at 72 hours	5(0.5–15)	10(8.5–20)	0.068
**Imaging Data**			
Infarct side (right),%	45.7	50.0	0.927
Occlusion pattern (TIMI 0/1-2/3),%	47.1 /47.9/5.0	66.7 /33.3/0.0	0.475
Recanalization (Present), %	70.7	66.7	0.803
ASPECTS	10(8–10)	9.5(8.5–10)	0.645
Admission CBV abnormality(mL)	62.25 (25.66–93.21)	56.90 (19.92–87.93)	0.462
Admission MTT abnormality(mL)	94.19 (45.58–120.13)	109.83 (42.52–123.18)	0.543
Admission penumbra,%	95.83	100	0.680
Collateral status (score 0/1-4)%	4.3/ 95.7	16.7/ 83.3	0.067
**Admission HPrs-PCT (cm**^**2**^**)**			
at 3 mL/100g/min	181.42 (40.98–326.58)	286.97 (106.69–561.30)	0.073
at 4 mL/100g/min	61.76 (15.69–186.06)	120.81 (53.39–327)	0.091
at 5 mL/100g/min	29.18 (6.97–104.21)	76.76 (36.51–204.05)	0.065
at 6 mL/100g/min	15.05 (4.47–52.42)	50.11 (18.51–124.47)	0.068
at 7 mL/100g/min	8.01 (1.83–26.48)	36.42 (12.23–65.46)	**0.039**
**Treatment and Outcome**			
Stroke onset to CT(min)	159(120–190.75)	150(124.5–213.75)	0.911
tPA dose(mg)	66(58.38–72)	63(56.75–70)	0.318
90-Day modified Rankin score	3(1–4)	4(2–4.25)	0.184

Unless otherwise specified, data are medians with interquartile ranges between parentheses. NIHSS, National Institutes of Health Stroke Score; TIMI, Thrombosis in Myocardial Infarction; rtPA, recombinant tissue plasminogen activator; ASPECTS, Alberta Stroke Programme Early CT score; CBV, cerebral blood volume; MTT, mean transit time; HPrs-PCT, high-permeability region size; HT, hemorrhagic transformation; PH, parenchymal hematoma; NA, not applicable.

### HPrs-PCT characteristics

HT was located in the region of large HP_rs_-PCT in all cases (Fig 1). Patients with HT had significantly larger HP_rs_-PCT than those without; this was true for all the analyzed thresholds (Table 1). However, differences in HP_rs_-PCT between patients with PH-2 and those with non-PH-2 were significant only at the 7mL/100g/min threshold (Table 1). The HP_rs_-PCT cutoffs decreased as the thresholds increased (Table 2). Sensitivity, specificity, and NPV were higher than PPV for all thresholds. Overall, the NPV of HP_rs_-PCT had the best performance (Table 2).

**Table 2:** Accuracy of HPrs-PCT thresholds in predicting hemorrhagic transformation.

	Area Under Curve (95%CI)	HPrs-PCT cut-off (cm^2^)	Sensitivity	Specificity	Positive Predictive Value	Negative Predictive Value
**Prediction Non HT vs any HT**						
at 3 mL/100g/min	0.659(0.572,0.745)	241.86	0.541	0.681	0.345	0.827
at 4 mL/100g/min	0.664(0.578,0.750)	281.36	0.570	0.933	0.556	0.804
at 5 mL/100g/min	0.693(0.609,0.777)	59.35	0.595	0.689	0.373	0.845
at 6 mL/100g/min	0.707(0.625,0.790)	56.29	0.486	0.815	0.450	0.836
at 7 mL/100g/min	0.712(0.630,0.795)	29.42	0.514	0.832	0.487	0.846
**Prediction PH-2 vs non PH-2**						
at 3 mL/100g/min	0.654(0.567,0.740)	492.39	0.333	0.896	0.211	0.942
at 4 mL/100g/min	0.626(0.538,0.714)	357.79	0.250	0.951	0.300	0.938
at 5 mL/100g/min	0.646(0.612,0.730)	116.50	0.333	0.792	0.118	0.934
at 6 mL/100g/min	0.665(0.579,0.751)	114.05	0.333	0.910	0.235	0.942
at 7 mL/100g/min	0.678(0.593,0.763)	91.83	0.350	0.938	0.250	0.938

### Prediction of HT

In the multivariate analysis, the best model for predicting HT (AUC 0.77) included admission HP_rs_-PCT at 7mL/100g/min, platelets, and baseline NIHSS (Table 3). However, the 7mL/100g/min HP_rs_-PCT threshold was the only predictor of PH-2 (OR1;AUC = 0.68,95%CI:1–1, p = 0.045). The sensitivity, specificity, PPV, and NPV of HP_rs_-PCT >91.83cm^2^ for the prediction of PH-2 were 35%, 93.8%, 25%, and 93.8%, respectively (AUC 0.678, 95%CI:0.593–0.763).

**Table 3:** Models selected from multiple regression analyses for predicting hemorrhagic transformation.

Any hemorrhagic transformation				
Independent Variable	β coefficient (x10^-3^)	Odds Ratio (95%CI)	p-value	AUC
*Univariate Analysis*				
HPrs-PCT at 7mL/100g/min	0.03	1.00(1.00–1.00)	<0.001	0.66
Admission MTT abnormality (mL)	0.01	1.00(1.00–1.00)	0.031	0.62
Admission NIHSS	99,41	1.10(1.04–1.17)	0.001	0.68
Platelets	-0.01	1.00 (1.00–1.00)	0.030	0.62
*Multivariate Analysis* Platelets	-0.01	1.00(1.00–1.00)	0.013	0.73
Admission NIHSS	111.86	1.11(1.05–1.19)	<0.001	
HPrs-PCT at 7 mL/100g/min	0.03	1.00 (1.00–1.00)	0.003	0.73
Admission NIHSS	85.93	1.07(1.01–1.13)	0.006	
HPrs-PCT at 7mL/100g/min	0.03	1.00(1.00–1.00)	0.004	0.77
Platelets	-0.01	1.00(1.00–1.00)	0.018	
Admission NIHSS	102.28	1.11(1.04–1.19)	0.003	
**PH-2**				
*Univariate Analysis*				
PSA at 7mL/100g/min	0.04	1.00(1.00–1.00)	0.045	0.68

## Discussion

In this study, we analyzed different thresholds for generating user-independent permeability maps in predicting HT after tPA administration in patients with MCA infarction. Our results suggest that HP_rs_-PCT at a threshold of 7mL/100g/min is highly specific for identifying patients with a low probability of developing HT after tPA administration. More importantly, HP_rs_-PCT at 7mL/100g/min lower than the defined cut-off value was the only independent predictor of PH-2 in our study and so, this method could be useful to improve safety and outcomes in patients with MCA infarction treated within the current 4.5-hour window.

HT in ischemic tissue after tPA administration is the result of a complex pathophysiologic process that involves BBB damage as well as many other factors [24–27]. In fact, in our multivariate regression analysis, the optimal model to predict HT (AUC = 0.77) included the HP_rs_-PCT threshold at 7mL/100g/min together with platelets and baseline NIHSS, and although HP_rs_-PCT at this threshold was the only predictor of PH-2, the sensitivity and PPV were low, making it necessary to consider other clinical and imaging parameters to avoid overestimating the risk of bleeding. Previously published data have shown that several parameters evaluated by CT perfusion might be useful for the prediction of HT. In this respect, lower CBV values [28,29] as well as higher MTT [6] and Tmax>14 seg [30] have been reported to be associated with intracranial hemorrhage in patients with acute ischemic stroke. In spite of this, the results cannot be considered as cleary consistent, since both MTT and CBV were included in these studies but the results in terms of prediction of bleeding were different depending on the study. According to this variability, in our study we have found lower CBV values in patients with HT as well as in patients with PH-2, although the differences were not significant. Regarding MTT, we have also found lower values in patients with HT and PH-2, but the values reached statistical significance only when the comparison was made between patients with and without HT.

Since HT is the result of BBB disruption, imaging techniques to characterize BBB status may be more useful in identifying patients who might safely benefit from these therapies. Predicting which patients will develop HT after tPA is a challenge and the quantification of BBB damage after stroke is probably essential in overcoming this challenge. In agreement with this idea, levels of biomarkers involved in the pathogenesis of BBB damage, such as matrix metalloprotease-9 and cellular fibronectin, have been demonstrated to be highly predictive of severe HT after tPA administration [26].

Accumulating evidence suggests that permeability imaging features might be good predictors of HT. However, the best BBB-permeability volume threshold to predict HT by PCT has not been established yet, probably due to differences in image acquisition and data modeling. Moreover, inclusion criteria among the patients studied were not homogeneus and the sample sizes were usually small. In fact, Lin et al. reported that any threshold between 4.99mL/100g/min (S = 100%; Sp = 80%) and 5.88mL/100g/min (S = 66.7%; Sp = 100%) can be used to predict HT after tPA administration. They used first-pass dynamic PCT and the study included 50 patients with acute ischemic stroke, 18 of whom had received tPA. Only six patients had HT, 3 in the group treated with thrombolysis [19]. In the study from Aviv et al., which included 41 patients with acute ischemic stroke, 22 treated with tPA, the best threshold for predicting HT was 0.23mL/100g/min (S = 77%; Sp = 94%). Twenty-tree patients developed HT in this study (15, HI and 8, PH). Permeability surface (PS) was higher in patients with HT compared to those without, but no differences were found between patients with HI and PH. On the other hand, PS was similar in patients with HT regardless of the administration of tPA [16]. With the objective of evaluating the predictive capacity of BBB permeability measurements on the development of sICH, Hom et al. analyzed a dataset of 32 patients with acute ischemic stroke by using delayed-adquisition PCT data and the Patlak model. Only 3 patients developed sICH. In spite of this small sample size, a threshold of 7mL/100g/min was found to be predictive of this complication (S = 100%; Sp = 79%) [15]. Ozkul-Wermester et al. [14] studied a total of 86 patients, 27 of whom developed HT. PS was significantly higher in patients with HT. The authors reported 96% NPV in predicting HT using a PS threshold>0.84ml/100g/min. In this study, the authors also found a poor collateral status to be associated with HT, a fact that is probably related to a more severe BBB disruption, since pial collateral arteries seem to help irrigate ischemic territory dowstream of the trombus [8,14]. According to this, a poor collateral status has also been demonstrated to be associated with HT in our study. More recently, Yen et al. evaluated the relative PS (rPS) area (ratio of the PS on the side of the acute ischemic stroke to the PS on the contralateral side) and compared this value in patients with and without HT. The study included 42 patients, 15 of whom had HT. Patients with a mean rPS of 1.3 had an increased likelihood of subsequent HT (S = 71.4; Sp = 78.6; PPV = 62.5; NPV = 84.6). However, no difference was found between petechial and parenchymal hemorrhagic events [13].

Our study included the biggest sample size previously studied with the objective of determining the capacity of HP_rs_-PCT in the prediction of intracranial bleeding after tPA treatment. Unless other studies, we only included patients treated with tPA because the increased risk of HT and the worse prognosis associated with this complication could be avoided if we were able to improve the selection of treated patients. Our results show that, although different thresholds did not differ significantly in their ability to predict any HT, the only independent predictor for PH-2 was the 7mL/100g/min threshold. In agreement with previously published data, patients with HT also had higher baseline NIHSS and larger ischemic lesions, as suggested by MTT maps [4,14].

However, in our study we have to acknowledge certain limitations. This is a single-center study that may not be representative of more diverse populations, thus precluding the generalization of our results. On the other hand, although we have shown that PCT-PCA maps seem to be useful for the prediction of PH-2 after tPA treament, the small simple size of sICH did not allow conclusions to be drawn in this subgroup of patients, a fact that is commom among previously published data. In fact, only one study has reported an association between PS and the development of sICH, but only 3 patients with sICH were evaluated [15]. As this particular subgroup of patients with intracranial bleeding are the most clinically relevant, larger cohorts of this group need to be assessed to clarify the usefulness of HP_rs_-PCT maps in predicting this complication. Reported permeability measurements in our study are different from those previously reported. The molecular size and charge of the contrast agent used, acquisition protocol (especially duration) and post-processing techniques, noise level, ROI location, infarct core/penumbra definitions, as well as the method used for permeability analysis, may be factors that might explain the difference in permeability values reported in the literature. Changes in the BBB during ischemia and reperfusion are dynamic and complex, so various thresholds could be used to process the permeability maps in clinical scenarios, and which would provide the best information remains to be determined. Standardized HP_rs_-PCT calculation software and further studies are needed to validate an optimal threshold to predict HT. In our study, we focused only on HP_rs_-PCT maps because they are quickly (<1 minute) and easily obtained after image acquisition with user-independent automatic segmentation and no need to draw regions of interest, making this approach feasible in acute settings. Nevertheless, models integrating other permeability indexes might provide more accurate predictions. Although 7mL/100g/min was found to be the optimal threshold to predict HT in our study, we cannot rule out that baseline permeability thresholds over the tested range or thresholds in our study may have a greater predictive capacity. Moreover, it has been reported that rPS measured in the infarct core with nonlinear regresssion (NRL) method has a superior discriminative power for the prediction of HT than *K*^trans^ measured with either Patlak analysis with a fixed offset or NLR, and conventional perfusion parameters. [31] Unfortunately, in our study we did not perform comparisons of the PS values between ipsilateral and contralateral areas, so we do not have rPS values to determine whether this parameter may have given us a better estimate of the hemorrhagic transformation risk. On the other hand, previous data have also shown that rPS does not seem to discriminate between less and more severe HT, [16] and so further studies are necessary in order to clarify the value of this parameter in the prediction of clinically meaningful HT.

## Conclusions

Our study demonstrates, in a large series of patients with MCA infarction, that HP_rs_-PCT can be useful in the prediction of intracranial bleeding after tPA administration with 4.5 hours of symptom onset. More specifically, HP_rs_-PCT can reliably identify patients at low risk of developing HT and PH-2. By using this approach, patients with low risk of bleeding beyond the current therapeutic window for tPA administration could potentially be identified and safely treated. Further studies are, however, necessary in order to define the most accurate thresholds for the prediction of bleeding and to determine how HP_rs_-PCT might refine clinical decision making in acute stroke patients treated within and beyond the current tPA therapeutic window.
